# Dead Bugs Don’t Mutate: Susceptibility Issues in the Emergence of Bacterial Resistance

**DOI:** 10.3201/eid0901.020172

**Published:** 2003-01

**Authors:** Charles W. Stratton

**Affiliations:** * Vanderbilt University School of Medicine, Nashville, Tennessee, USA

**Keywords:** Drug resistance, microbial, respiratory tract infections, pathogenicity, ketolide, therapeutic use

## Abstract

The global emergence of antibacterial resistance among common and atypical respiratory pathogens in the last decade necessitates the strategic application of antibacterial agents. The use of bactericidal rather than bacteriostatic agents as first-line therapy is recommended because the eradication of microorganisms serves to curtail, although not avoid, the development of bacterial resistance. Bactericidal activity is achieved with specific classes of antimicrobial agents as well as by combination therapy. Newer classes of antibacterial agents, such as the fluoroquinolones and certain members of the macrolide/lincosamine/streptogramin class have increased bactericidal activity compared with traditional agents. More recently, the ketolides (novel, semisynthetic, erythromycin-A derivatives) have demonstrated potent bactericidal activity against key respiratory pathogens, including *Streptococcus pneumoniae, Haemophilus influenzae, Chlamydia pneumoniae*, and *Moraxella catarrhalis*. Moreover, the ketolides are associated with a low potential for inducing resistance, making them promising first-line agents for respiratory tract infections.

As the 19th century drew to a close, the work of Joseph Lister ushered in the antimicrobial era. Lister was among the first scientists to appreciate the implications of Pasteur’s theory that microorganisms are involved in human disease (*1*). Accordingly, he examined the inhibitory effect of various chemicals on the growth and viability of bacteria and directly applied the results to the practice of medicine by using phenol (as well as heat) to sterilize surgical instruments. After this early example of infection control through antisepsis, the next step was inevitable: when chemicals with antibacterial activity were discovered, they were soon used in the treatment of infected patients. The ensuing clinical success was so dramatic that these agents were hailed as miracle drugs. By the second half of the 20th century, the practice of medicine enjoyed almost complete dominance over infectious bacteria (*2*).

Ironically, these same miraculous drugs now jeopardize the miracle, as evidenced by the widespread emergence of antibacterial resistance in the last decade (*3*–*7*). For example, methicillin-resistant *Staphylococcus aureus* strains have recently appeared in community-acquired infections (*8*), and *Streptococcus pneumoniae* strains resistant to both penicillins and macrolides (the antibacterial agents used most frequently for pneumococcal infections) have become prevalent throughout the world. Indeed, rates of *S. pneumoniae* resistance to penicillin now exceed 40% in many regions, and a high proportion of these strains are also resistant to macrolides. Moreover, the trend is growing rapidly. Whereas 10.4% of all *S. pneumoniae* isolates were resistant to penicillin and 16.5% resistant to macrolides in 1996, these proportions rose to 14.1% and 21.9%, respectively, in 1997 (*9*). A more recent susceptibility study conducted in 2000–2001 showed that 51.5% of all *S. pneumoniae* isolates were resistant to penicillin and 30.0% to macrolides (*10*).

The urgent need to curtail proliferation of antibacterial-resistant bacteria has refocused attention on the proper use of antibacterial agents. That the use of any antibacterial agent or class of agents over time will result either in the development of resistance to these agents or in the emergence of new pathogenic strains that are intrinsically resistant is now widely accepted. An example of the development of resistance is the mutation of *S. pneumoniae* to produce a multidrug-resistant strain (*11*). An example of a new resistant pathogenic strain is exemplified by the emergence of *Enterococcus gallinarum* as a nosocomial pathogen due to its intrinsic resistance to vancomycin (*12*). Keeping these phenomena in check requires a comprehensive strategy that includes, whenever possible, the selection of antibacterial agents in dosages sufficient to be bactericidal (*13*). A bactericidal effect is desired because, to put it succinctly, dead bugs don’t mutate. In other words, if microbial pathogens causing infection are killed by antimicrobial therapy, rather than inhibited, mutations that might already exist or occur under the selective pressure of the antimicrobial agent are less likely to be promulgated. This principle will be briefly reviewed in relation to respiratory tract infections.

## Clinical Relevance of Bacteriostatic versus Bactericidal Activity

All of the effects of antimicrobial agents against microbes, including the delineation of microbial resistance, are based upon the results of in vitro susceptibility testing. Most of these susceptibility tests only measure bacteriostatic activity even though the agent being tested may have bactericidal activity. Thus, the clinical relevance of susceptibility testing itself could be questioned. Numerous authors have extensively reviewed this issue over the years (*14*–*16*). These authors point out the paucity of studies that have critically evaluated the effectiveness of antimicrobial therapy with results of in vitro susceptibility tests. Such critical evaluations are not easily done, as susceptibility tests do not take into account the normal host defense mechanisms. However, the detection of resistance is somewhat predictive of poor outcome, although in the normal host this may be less clinically important due to the interaction of host defenses (*17*,*18*).

The ability of bactericidal activity to influence therapeutic efficacy and clinical outcome has been evaluated in infections that typically are refractory to antimicrobial therapy. These infections include endocarditis, meningitis, osteomyelitis, and infections in the neutropenic host. All are similar in that antimicrobial penetration and host defense mechanisms at the site of infection are limited. Both experimental models of infection (*19*–*22*) and clinical studies (*23*–*27*) have shown that bactericidal activity predicts therapeutic efficacy and results in improved clinical outcome. Bactericidal activity has been considered less important in respiratory tract infections, with the exception being acute infectious exacerbations in cystic fibrosis (*28*). However, the relevance of pharmacokinetic and pharmacodynamics in the selection of antibiotics for respiratory tract infection has become increasingly recognized (*29*). Issues such as drug concentration at the site of infection, bactericidal activity, postantibiotic effect, and duration of therapy needed to achieve these effects are now being considered when antimicrobial agents are selected for the therapy of respiratory tract infections (*29*,*30*).

Although host factors may allow a bacteriostatic agent to be used successfully in an infected patient, these factors appear to be less able to curtail the emergence of resistance. Resistance, as a rule, occurs more rapidly with bacteriostatic agents such as tetracyclines, sulfonamides, and macrolides than it does with bactericidal agents such as beta-lactams and aminoglycosides (*31*–*33*). An example of this can be seen with *S.* *pneumoniae*. Beta-lactam agents have been the antimicrobial agents of choice for the therapy of pneumococcal pneumonia since penicillin was first clinically introduced in the 1940s (*17*). Penicillin resistant strains of *S.* *pneumoniae* have taken more than 4 decades to emerge. The emergence of macrolide resistance in *S. pneumoniae* has been rapid in comparison and has even been described during treatment of pneumococcal pneumonia (*34*). Bactericidal activity thus may be useful in the therapy of respiratory tract infections as a means to curtail, but not avoid, the emergence of resistance.

## Antibacterial Resistance Mechanisms: Bactericidal versus Bacteriostatic Activity

The key to resolving the problem of antibacterial resistance lies in identifying the mechanisms that engender it (*31*–*33*). Among the most important mechanisms are decreased ability of antibacterials to penetrate the bacterial cell wall, active efflux of antibacterial agents, inactivation of antibacterial agents, destruction of antibacterial agents, alteration of antibacterial target sites, development of bypass pathways around antibacterial targets, and constitutive phenotypic variation in bacterial physiology.

Fundamental to many of these mechanisms is mutation of bacterial DNA. Subsequent exposure of the microorganism to a specific agent may then select the mutant, leading to the emergence of resistance. Some resistance mechanisms, such as bacterial production of beta-lactamase, are inducible or can be derepressed (*35*), requiring either upregulation or mutation of genetic material. Thus, if resistance is to be suppressed, the opportunity for bacterial upregulation or mutation of genetic material must be minimized.

One way to minimize upregulation, mutation, or both is by using bactericidal rather than merely bacteriostatic agents. Microorganisms inhibited by a bacteriostatic agent or exposed to an insufficient concentration of a bactericidal agent remain alive and, ipso facto, retain the potential to become resistant or promulgate any resistance selected by the exposure to the antimicrobial agent. An example of this principle can be seen with the upper respiratory tract pathogen, *Streptococcus pyogenes,* which to date has not developed resistance to penicillin, a bactericidal agent, but has developed resistance to erythromycin, a bacteriostatic agent (*36*–*38*). Erythromycin resistance in *S. pyogenes* largely is due to upregulation of efflux (36) or to ribosomal mutation (*37*). Antimicrobial agents that kill this pathogen should be less likely to promulgate any strains having such resistance. Another example is seen with the *omp* genes of gram-negative microorganisms. These *omp* genes encode porins that are sometimes flanked by insertion sequences. In the presence of the bacteriostatic agent, the mobility of insertion sequence–flanking *omp* genes can be attenuated and will result in disruption of the *omp* genes. The reduced expression of these porins may lead to reduced uptake of the inducer, the antibacterial agent. Specifically, insertion sequence interruption of the *ompK36* porin gene in respiratory tract pathogen *Klebsiella pneumoniae* has been shown to interfere in the expression of this porin gene and has resulted in clinical failure (*39*). If a bactericidal agent kills a pathogen such as *Klebsiella* before mutation of the porin gene, resistance is less likely to develop. These two examples illustrate the desirability of achieving bactericidal activity to curtail the emergence of resistance.

## Bactericidal Activity Achieved by Combination Therapy

Bactericidal activity can be achieved through the mechanism of action for a single antimicrobial agent or by the use of combination therapy, or both. Sulfamethoxazole/trimethoprim (SMX-TMP) is an example of a combination of two agents, each of which alone is bacteriostatic, that achieves bactericidal activity. Sulfamethoxazole inhibits dihydropteroate synthase, the bacterial enzyme that catalyzes the incorporation of *p-*aminobenzoid acid into dihydropteroic acid, the immediate precursor of folic acid, while trimethoprim was specifically synthesized as an inhibitor of dihydrofolate reductase (*40*). SMX-TMP has long been used for the therapy of respiratory tract infection (*41*) and has proven particularly useful in the treatment of acute exacerbations of chronic bronchitis. In fact, the World Health Organization continues to be recommend SMX-TMP as the first-line treatment for pneumonia in children because of its low cost and ease of dosing. Resistance to SMX-TMP has emerged more slowly than for either agent used alone (*42*). However, emergence of resistance to *S. pneumoniae* has occurred (*43*) and now may limit the use of SMX-TMP in respiratory tract infections.

Combinations of antimicrobial agents are also used in the therapy of bacterial endocarditis to achieve synergism leading to increased bactericidal activity and improved sterilization of infected valves. Bactericidal synergy for *S. epidermidis* can be demonstrated in vitro for the combination of vancomycin, rifampin, and gentamicin, which correlates well with the therapeutic results in an experimental animal model (*44*). In a comparable clinical study of patients with prosthetic valve endocarditis caused by *Staphylococcus* *epidermidis*, 90% were cured with a combination of vancomycin, rifampin, and/or gentamicin, compared with only 50% cured among those receiving vancomycin alone (*45*). Combination therapy for respiratory tract infections is less well studied except for acute respiratory tract infections occurring in cystic fibrosis patients. For example, combination therapy for treatment of *Pseudomonas aeruginosa* pulmonary infections in cystic fibrosis patients achieved a cure rate of 89% if peak serum bactericidal titers were >1:128 (*28*). In contrast, 100% of patients failed therapy if their peak serum bactericidal titers were <1:16.

## Bactericidal Activity Achieved with Novel Bactericidal Agents

### Fluoroquinolones

Other examples of the importance of bactericidal activity are the fluoroquinolones. Studies of the bactericidal action of the quinolones against *Escherichia coli* demonstrate at least two independent and important mechanisms of action. First, all quinolones exert bactericidal action by inhibiting topoisomerases. These bactericidal agents are only effective if the bacteria are actively dividing or synthesizing proteins and mRNA. The bactericidal activity of the quinolone nalidixic acid, for example, is minimized by chloramphenicol, which prevents protein synthesis, and by rifampin, which prevents RNA synthesis. However, ciprofloxacin and ofloxacin/levofloxacin respond differently. Although the bactericidal action of these fluoroquinolones against *E. coli* is reduced, bactericidal action is not entirely eliminated by chloramphenicol or rifampin. This lack of elimination of bactericidal action suggests that these agents possess a secondary bactericidal mechanism of action that does not depend on the synthesis of protein and RNA, and that may be active when the bacteria are in a nonreplicating state (*46*).

To understand this secondary bactericidal effect, consider the bacterial inducible SOS system (*47*). Consisting of approximately 20 genes, this system repairs structural damage to DNA caused by antibacterial agents, mainly through bypass repair (*46*–*50*). This mechanism tends to be error prone and often leads to mutants. Another effect of the SOS response, activated by fluoroquinolone-induced damage to the bacterial DNA, is the discontinuation of cell replication. The organism can refrain from replication for only so long before it dies.

In addition to high concentrations of fluoroquinolone, which trigger the secondary bactericidal mechanism, higher concentrations at DNA targets also play a role in the emergence of resistance because the postantibiotic effect of the fluoroquinolones is dependent upon concentration, time, and the microorganism. If the concentration of fluoroquinolone attained at the bacterial DNA targets is high enough to activate the SOS system for a duration that exceeds the capability of the particular microorganism to repair its DNA damage and replicate, the microorganism dies. No postantibiotic effect occurs, per se, since no microorganisms survive. If the fluoroquinolone concentration is not adequate, however, a race occurs between cumulative damage over time and the selection of a resistant mutant.

The concentration of fluoroquinolone required for SOS-mediated discontinuation of cell replication is expressed as a peak concentration/minimum inhibitory concentration (MIC) ratio and appears to require a ratio of approximately 10:1 (*50*,*51*). A rat model for *Pseudomonas* sepsis demonstrated that peak concentration/MIC ratios >20:1 once per day produced significantly (p<0.5) better survival—which may result in the selection of a mutant with altered topoisomerase—than did regimens using the same dosage on a more fractionated schedule (*52*). Dosages that led to peak concentration/MIC ratios <10 times the MIC did not result in as high a survival rate. Indeed, when the peak concentration/MIC ratio was <10 times the MIC, the best survival was predicted by the area under the curve/MIC ratio, since repeated exposure to the fluoroquinolone causes damage cumulatively. The length of time that fluoroquinolone levels in plasma exceeded the MIC had no influence on survival.

The emergence of fluoroquinolone resistance with respect to *Staphylococcus aureus* and *P. aeruginosa* has been well-documented (*53*). This major problem is due to a wide variety of fluoroquinolone-resistance mechanisms (*54*,*55*), particularly the mutation of DNA gyrase (*56*). While this type of resistance generally results in MICs only four- to eight-fold higher than the susceptible isolate, recent studies have reported the development of high-level resistance (e.g., ciprofloxacin MIC for *P. aeruginosa* of 1,024 mg/L) mediated by efflux pumps targeting multiple antibacterial agents (*57*,*58*). These multidrug efflux pumps could be overcome by high fluoroquinolone concentrations, some of which, however, would not be clinically achievable.

A rabbit meningitis model further demonstrates how the inability to achieve peak concentration/MIC ratios >10:1 influences the postantibiotic effect. In an in vivo study, an exposure to ciprofloxacin at the MIC had minimal impact (*59*), underscoring the value of bactericidal activity with respect to fluoroquinolone therapy. The greater the activity of the fluoroquinolone, the more likely the agent will achieve serum or tissue levels that are >10 times the MIC, which in turn determines the secondary bactericidal and postantibiotic effects. Consequently, newer fluoroquinolones such as gemifloxacin (*60*) and others now under development have markedly increased activity compared with traditional agents. For example, ciprofloxacin has MICs against *Streptococcus pneumoniae* of approximately 0.5 mg/L, while gemifloxacin has MICs of approximately 0.03 mg/L.

An important issue associated with the use of fluoroquinolones in the therapy of respiratory tract infections is the fact that fluoroquinolones also are used to treat other infections. The use of these agents for other infections means that the population already had been exposed to fluoroquinolones before their widespread use in respiratory tract infections. Exposure of normal flora in these patients to subbactericidal concentrations of fluoroquinolones may allow resistant strains to emerge. Cross-resistance is a well-recognized problem with fluoroquinolones (*55*), and the enormous prior exposure of the population to these agents may have created resistant strains in the normal flora of the mucosal surfaces, skin, gastrointestinal tract, and reproductive tract. In addition, prior exposure may result in increasing MICs due to subtle mutations of topoisomerases, which then may leave the microorganism only one step from a mutation that will produce overt resistance (*55*). An example of such subtle topoisomerase mutation is seen with fluoroquinolones such as levofloxacin, which have been recommended and widely used for the therapy of pneumococcal pneumonia when penicillin resistance to *S. pneumoniae* is a problem (*61*). Unfortunately, the population has had considerable prior exposure to earlier fluoroquinolones, which has allowed rapid emergence of fluoroquinolone resistance in *S.* *pneumoniae* (*62*). Failure of treatment of pneumococcal pneumonia due to resistance to levofloxacin recently has been described (*63*). This example confirms the problem of cross-resistance and further mutations resulting in increased resistance and suggests that newer fluoroquinolones such as gemifloxacin may be less effective or even ineffective against *S. pneumoniae.*

## Macrolides, Lincosamides, and Streptogramins (MLS)

Another class of antibacterials containing newly developed bactericidal agents are the macrolides. The term macrolide was originally applied to specific compounds produced by various *Streptomyces* species containing, as part of their structure, a macrocyclic lactone to which various deoxy sugars are attached. These bacteriostatic compounds bind to bacterial 50S ribosomes, inhibiting protein synthesis without a concomitant inhibition of nucleic acid synthesis. The classification has since been modified to include other structurally diverse agents, such as lincosamides and streptogramins, which are also produced by *Streptomyces* species and target the 50S ribosome. The term MLS (macrolide, lincosamide, and streptogramin) has become the accepted nomenclature for this important group of antibacterial agents—except when the emphasis is on structural similarity, in which case the erythromycin congeners (erythromycin-A, clarithromycin, and azithromycin) are often referred to as the “true macrolides” (*64*). Although antibacterial agents in the MLS class have been largely bacteriostatic, newer members demonstrate bactericidal activity. These new agents include quinupristin/dalfopristin and telithromycin.

The bactericidal mechanism of action of quinupristin/dalfopristin, a combination of two streptogramins, is unique (*65*). Dalfopristin is an olefinic macrolactone that binds to the 50S subunit of the prokaryotic ribosome and interferes with the function of peptidyl transferase, thereby inactivating the donor and acceptor sites of the ribosome. In addition, dalfopristin triggers a conformational change in the ribosome that greatly increases the affinity of quinupristin, a peptidic macrolactone, which also binds to the 50S subunit of the ribosome and halts peptide chain elongation. Consequently, protein synthesis is not only halted transiently by either component used alone but also halted permanently by the two components in combination, resulting in synergistic and concentration-independent bactericidal activity against many pathogens. This binding of both macrolactones distinguishes quinupristin/dalfopristin from other antibiotic classes (*66*,*67*), with the attendant prolongation of the postantibiotic effect (*68*) representing a distinct advantage over older agents (*69*). Quinupristin/dalfopristin is bactericidal against staphylococci and streptococci such as *S. pneumoniae*, generally bacteriostatic against *Enterococcus faecium,* and inactive against *E. faecalis* (*70*). Because quinupristin/dalfopristin is available only in an intravenous formulation, its utility for treating respiratory tract infections is limited to hospitalized patients. Moreover, clinical data on quinupristin/dalfopristin therapy of pneumococcal pneumonia caused by macrolide resistant strains of *S. pneumoniae* is lacking.

## Ketolides: Telithromycin

Telithromycin, the first of a new class of antibacterials, the ketolides, is approved for use in Europe and is currently being reviewed by the U. S. Food and Drug Administration (*71*). The clinical use of telithromycin in Germany, as well as safety data presented to the Food and Drug Administration, suggests that the toxicity and adverse reactions are similar to those of clarithromycin. This similarity is not surprising, as the ketolides are novel semisynthetic erythromycin-A derivatives structurally similar to clarithromycin. The C6-hydroxyl of erythromycin-A has been replaced by a methoxy group, as in clarithromycin, improving acid stability. The main structural innovation is the lack of the neutral sugar, cladinose, in position C3. The 3-l-cladinose sugar moiety is removed, and the resulting 3-hydroxy group is oxidized to a 3-keto group, which is responsible for preventing induction of macrolide resistance (*72*). Telithromycin is produced through substitution at positions 11 and 12 of the erythronolide A ring with a butyl imidazolyl pyridinyl side chain. The resulting C11,C12 carbamate extension facilitates a distinctly different and more effective interaction with domain II of the 23S rRNA than occurs with erythromycin-A or clarithromycin (*73*) and is responsible for increased activity against erythromycin-A–resistant gram-positive cocci, such as *S. pneumoniae*, which develop resistance due to increased efflux (*74**,**75*). Moreover, the bactericidal action is effective against a number of other key respiratory pathogens, such as *H. influenzae* and *C. pneumoniae* (*76*–*78*), other gram-negative bacilli, such as *M. catarrhalis* and *Bordetella pertussis,* and the intracellular pathogen *Legionella pneumophila* (*79*).

Unlike erythromycin-A (which interacts with bacterial 23S ribosomal RNA by contacts limited to hairpin 35 in domain II of the ribosomal RNA and to the peptidyl transferase loop in domain V^49^), telithromycin is not a true macrolide because the l-cladinose moiety at position C3 has been replaced by a keto group and by alkylaryl side chains at positions C11,C12. Although both erythromycin-A and telithromycin bind to the peptidyl transferase loop (the site of methylation by resistant bacteria), telithromycin binds much more avidly to hairpin 35 than erythromycin-A. In fact, telithromycin interacts strongly with two domains of the bacterial 23S rRNA (domains II and V), which fold together in the tertiary 23S rRNA to form a single drug-binding pocket (*80*,*81*). The lack of the L-cladinose moiety, as well as the enhanced binding at domain II and V, may explain why ketolides are associated with a low potential for inducing resistance (*82*,*83*) and contributes to telithromycin’s sustained activity against MLS_B_-resistant strains (in particular those with domain V modifications) (*73*). These features, in addition to the MIC and the amount of drug delivered to the infection site, are considered strong predictors of a positive outcome (*29*,*30*). However, the population has had enormous exposure to earlier macrolides. This exposure has an influence on the normal flora of mucosal surfaces. This influence means that resistance due to efflux or methylation of the 23S ribosome (domain V) may have already occurred in a large number of pneumococcal isolates. Macrolide-resistant strains of *S. pneumoniae* to date have had a low incidence of cross-resistance to telithromycin (*82*,*83*). However, increased efflux or other mutations might result in resistance to ketolides. To date, only two ketolide-resistant strains of *S. pneumoniae* have been identified (*84*) in over 10,000 pneumococcal isolates screened by the PROTEKT study (*10*). The MIC of one of these isolates was 1 mg/L; the other was 256 mg/L. Clearly, careful monitoring for ketolide resistance by surveillance studies such as the PROTEKT study will need to be continued.

## Conclusion

Meeting the challenge presented by the increasing numbers of bacterial pathogens resistant to common antibiotic treatments will require new types of antibacterial agents. Therapies that maximize bactericidal effects are important because they reduce the development of bacterial resistance mechanisms. Therefore, the use of bactericidal agents such as telithromycin for therapy of respiratory tract infections may well ensure that the antibacterial era endures long into the 21st century. However, careful monitoring of resistance will be needed to ensure that this agent remains active against common pathogens.

Stratton is associate professor of pathology and medicine and director of the Clinical Microbiology Laboratory at the Vanderbilt University Medical Center in Nashville, Tennessee. He is also editor of Antimicrobics and Infectious Diseases Newsletter. His research interests include the mechanisms of antimicrobial activity, antimicrobial resistance, and the pathogenesis of *Chlamydia pneumoniae.*
